# Association of nutritional status and serum albumin levels with development of toxicity in patients with advanced non-small cell lung cancer treated with paclitaxel-cisplatin chemotherapy: a prospective study

**DOI:** 10.1186/1471-2407-10-50

**Published:** 2010-02-21

**Authors:** Oscar Arrieta, Rosa M Michel Ortega, Geraldine Villanueva-Rodríguez, Maria G Serna-Thomé, Diana Flores-Estrada, Consuelo Diaz-Romero, Cindy M Rodríguez, Luis Martínez, Karla Sánchez-Lara

**Affiliations:** 1 Medical Oncology Department Instituto Nacional de Cancerología Av San Fernando No 22, Col Sección XVI, Mexico City, 14080 Mexico; 2 Medical Nutrition Department Instituto Nacional de Cancerología Av San Fernando No 22, Col Sección XVI, Mexico City, 14080 Mexico; 3 Instituto Nacional de Enfermedades Respiratorias (INER) Calzada de Tlalpan 4502, Col Sección XVI, Mexico City, 14080 Mexico; 4 Diana Laura Riojas de Colosio Oncology Center, Medica Sur Clinic and Foundation Puente de Piedra 150 Toriello Guerra, Mexico City, 14050 Mexico

## Abstract

**Background:**

A frequent manifestation of advanced NSCLC is malnutrition, even though there are many studies which relate it with a poor survival, its relation with toxicity has not yet been consistently reported. The aim of this study was to associate malnutrition and albumin serum levels with the occurrence of chemotherapy-induced toxicity in cisplatin plus paclitaxel chemotherapy-treated NSCLC.

**Methods:**

We prospectively evaluated 100 stage IV NSCLC patients treated with paclitaxel (175 mg/m^2^) and cisplatin (80 mg/m^2^). Malnutrition was assessed using SGA prior treatment. Neutrophil Lymphocyte Ratio (NLR) and the Platelet Lymphocyte Ratio (PLR) were used to determine the presence of systemic inflammatory response (SIR) and were related to the development of toxicity. Toxicity was graded according to NCI CTCAE version 3.0 after two chemotherapy cycles.

**Results:**

Median age was 58 ± 10 years, 51% of patients were malnourished, 50% had albumin ≤3.0 mg/mL. NLR ≥ 5 was associated with basal hypoalbuminemia (mean ranks, 55.7 vs. 39 p = 0.006), ECOG = 2 (47.2 vs. 55.4 p = 0.026) and PLR ≥ 150 were significantly related with a basal body mass index ≤20 (56.6 vs. 43.5; p = 0.02) and hypoalbuminemia (58.9 vs. 41.3; p = 0.02). Main toxicities observed after 2 cycles of chemotherapy were alopecia (84%), nausea (49%), neuropathy (46%), anemia (33%), lymphopenia (31%), and leukopenia (30%). Patients malnourished and with hypoalbuminemia developed more chemotherapy-induced toxicity overall when compared with those without malnutrition (31 vs 22; *p *= 0.02) and normal albumin (mean ranks, 62 vs 43; *p *= 0.002), respectively. Hypoalbuminemia was associated with anemia (56 vs 47; *p *= 0.05), fatigue (58 vs 46; *p *= 0.01), and appetite loss (57.1 vs 46.7; *p *= 0.004) compared with normal albumin. PLR ≥ 150 was related with the development of toxicity grade III/IV (59.27 vs. 47.03 p = 0.008) and anemia (37.9 vs 53.8 p = 0.004).

**Conclusion:**

SIR parameters were associated with malnutrition, weight loss and hypoalbuminemia. Chemotherapy-induced toxicity in NSCLC patients treated with paclitaxel and cisplatin was associated with malnutrition and hypoalbuminemia. Early nutritional assessment and support might confer beneficial effects.

## Background

Non-small-cell lung cancer (NSCLC) is highly prevalent and the major cause of cancer-related deaths in the U.S., Mexico, and worldwide [1-3]. NSCLC represents approximately 80% of lung neoplasms. While surgery is the principal curative option, fewer than 20% of patients have resectable disease, permitting their being operated at time of diagnosis [4]. Multiple studies have clearly shown that chemotherapy is important in the palliative care of advanced NSCLC, with objective tumor-response percentages of 25-35%; significant extension of overall survival has also been obtained when compared with the best support treatment. One of the most frequently employed first-line regimens consists of cisplatin (P) plus paclitaxel (TXN) [5].

P is widely used as part of the treatment of various malignant neoplasms, such as ovary, breast, lung, and head and neck cancer [6,7]. Further improvements in survival have been achieved when it is combined with third-generation agents [8,9]. TXN has the ability to block cells in the late G2 phase of the cell cycle by microtubule polymerization and stabilization [10]. TXN is transported by binding with plasma proteins, such as albumin, the latter significant for its pharmacokinetic behavior; it is also extensively protein-bound (95-98%) to tissue proteins [11], and its removal is primarily carried out by hepatic metabolism [12].

Despite efforts to diminish treatment-related toxicity and to preserve or improve patients' quality of life (QOL), all combinations of platin and third-generation agents including their modifications maintain a percentage of adverse events [13]. The most common hematologic adverse effects comprise leukopenia and anemia, while the most frequently found non-hematologic adverse reactions are nausea and vomiting. Peripheral neuropathy, arthralgias, myalgias, and dermatologic toxicity can be encountered less frequently, rendering dose reduction necessary [14-16].

The prevalence of malnutrition-associated morbidity in patients with cancer ranges from 40-80% [17]. The process of nutritional and functional decline in the patient with cancer is so common that it is often accepted as part of the disease itself and its treatment. Currently, there is evidence that the presence of a systemic inflammatory response (SIR) is associated with increased weight loss, an elevated resting energy expenditure, loss of lean tissue and functional decline [18]. Malnutrition is associated with a higher risk of developing complications and with mortality, sometimes lengthening the hospital stay by up to 90%, thus increasing hospitalization costs by 35-75% [19]; malnutrition is also related with a reduction in response [20,21], hence influencing QOL of patients, their sense of well-being, and emotional aspects. Malnutrition is related with hypoalbuminemia, and both are common in patients with NSCLC [22], especially in non-developed countries, where malnutrition is prevalent in itself. Currently, there are certain clinical and biochemical measurements utilized in the nutritional evaluation of oncologic patients, such as the Subjective Global Assessment (SGA) [23], as well as the albumin serum levels, which reflects both the loss of the amount of lean tissue and systemic inflammatory response [18,24-26]. Neutrophil Lymphocyte Ratio (NLR) and the Platelet Lymphocyte Ratio (PLR) have also been demonstrated as indicators of systemic inflammatory response [18]. Indeed, the magnitude of the increase PLR and NLR has been shown to be associated with poorer survival in patients with cancer, particularly in patients with advanced disease [27-30]. The aim of this study was to associate malnutrition and albumin serum levels with the occurrence of chemotherapy-induced toxicity in P plus TXN treated NSCLC.

## Methods

### Study population

With previous institutional research and ethics board approval, we conducted a prospective analytical study at the Instituto Nacional de Cancerología in Mexico City. All patients gave written informed consent. We evaluated 100 consecutive patients recently diagnosed with NSCLC who attended the Thorax Neoplasms Clinic from January 2007 to February 2009. Inclusion criteria were as follows: patients with a diagnosis of stage IV histopathological or cytological NSCLC, with Eastern Cooperative Oncology Group (ECOG) score of ≤2, aged 18-80 years, and eligibility to receive TXN (175 mg/m^2^) and P (80 mg/m^2^) based first-line palliative chemotherapy every 3 weeks for at least two cycles. Exclusion criteria included patients who declined participation in the study, who required biochemical or hematological alteration-related P or TXN dose reduction, who had poor functional state, those who had received previous treatment (surgical, radiotherapy, and/or chemotherapy), and patients with signs of infection, acute inflammatory processes, or liver disorders.

### Procedures

Prior to chemotherapy administration, clinical, biochemical, and nutritional evaluations by means of Subjective Global Assessment (SGA) were conducted. Initial values were collected in a database that also included patient characteristics. All patients received chemotherapy at the institution with the same premedication drugs (aprepitant day 1: 125 mg; 2^nd ^and 3^rd ^days, 80 mg PO, ondansetron 16 mg IV, dexamethasone 16 mg IV, chlorphenamine 10 mg IV, ranitidine hydrochloride 50 mg IV) and hydration.

The biochemical evaluation comprised complete blood count (including lymphocytes), blood chemistry (glucose, BUN, and creatinine), and liver function tests (AST, ALT, total and direct bilirubin, total proteins, albumin, and globulin). Venous blood samples were drawn from patients after an overnight fast. All laboratory values were determined using routine automated analyzers at the Department of Clinical Chemistry of the Instituto Nacional de Cancerología. Serum albumin levels were measured with the bromcresol purple method and employed as the biochemical marker of nutritional status: levels <3.5 mg/dL of albumin (hypoalbuminemia) were taken to indicate malnutrition; in addition, levels <3.8 mg/dL were considered for analysis, given that this is the lower limit used at the Institute. The NLR was defined as the absolute neutrophil count divided by the absolute lymphocyte count. The PLR was defined as the absolute platelet count divided by the absolute lymphocyte count. Both the NLR and PLR were calculated from the full blood count routinely performed one day before the first cycle of chemotherapy: NLR ≥5 and PLR ≥150 were taken to indicate SIR as previously described [18,27-30].

Baseline nutritional status evaluation was performed by three Clinical Nutritionists (CNs) (KS, GS, and ST) and consisted of questionnaires utilized for detection of undernourishment, anorexia, hunger-satiety sensations, and SGA; the CNs were blinded to the toxicity evaluation. SGA consists of a brief nutritional history (weight loss during the last 6 months, dietary changes, and a short physical examination of subcutaneous fat, muscle mass, albumin serum levels and fluid balance). SGA classifies patients as having severe or moderate malnourishment, or being well-nourished.

The clinical evaluation included ECOG, weight, height, and body mass index (BMI). Body weight was measured, in light clothing and without shoes, to the nearest 0.10 kg. Height was measured to the nearest 0.5 cm. BMI was calculated as weight (kg)/height squared (m^2^). BMI <20 is widely accepted as indicating that the subject is underweight, particularly in well-developed countries, and 18.5 is recommended as a practical lower limit for the majority of populations. Therefore, a diagnosis of malnutrition was made when BMI was <18.5 kg/m^2^.

After two chemotherapy cycles were received, biochemical evaluation was repeated and signs and symptoms of toxicity were evaluated; these were graded according to National Cancer Institute Common Terminology Criteria of Adverse Events (NCI CTCAE) (version 3.0).

### Statistical analysis

For descriptive purposes, continuous variables were summarized as arithmetic means, medians, and standard deviations (SDs), while categorical variables were expressed as proportions and confidence intervals (CIs). Inferential comparisons between groups (changes in biochemical values) were carried out using the Student *t *or the Mann-Whitney *U *test, according to data distribution determined by the Kolmogorov-Smirnov test. The chi squared or Mann-Whitney *U *test were utilized to associate clinical and biochemical parameters, and malnutrition. Statistical significance was determined as *p *< 0.05 with a two-sided test. SPSS software package version 15 (SPSS, Inc., Chicago, IL, USA) was employed to analyze the data.

## Results

General characteristics of all patients are shown in Table 1. Median age was 58.9 ± 10.5 years and mean BMI was 24.9 ± 3.7 kg/m^2^. Forty nine patients were well-nourished at the time of SGA application (A) and malnourishment was found in the remaining 51%: 34% of patients were moderately malnourished (B) and 17% were severely malnourished (C). The range of albumin serum levels found in our patients was 2.5-4.3 mg/dL, with a mean of 3.1 ± 0.5 mg/dL, which is below the standard level of 3.5 mg/dL. The median NLR and PLR were 3.5 and 225.7, respectively. NLR ≥ 5 was associated with baseline hypoalbuminemia (55.7 vs. 39 p = 0.006), ECOG = 2 (47.2 vs. 55.4 p = 0.026). PLR ≥ 150 was significantly related with a basal BMI ≤20 (56.6 vs. 43.5; p = 0.02) and hypoalbuminemia (58.9 vs. 41.3; p = 0.02).

**Table 1:** Clinical characteristics of the patients

Variable	n = 100
Gender	
Male	53%
Female	47%

Age years (mean ± sd)	58.9 ± 10.6

ECOG	
0	8%
1	65%
2	27%

BMI (kg/m^2^)	
Mean ± sd	24.9 ± 3.7
Median (range)	24.5 (15.9-35)

SGA	
A	49%
B	34%
C	17%

Albumin (mg/dL)	
Mean ± sd	3.1 ± 0.5

Diabetes at diagnosis	18%

NLR	
Median (range)	3.5 (.32-34.3)

PLR	
Median (range)	225.7 (16.2-843.3)

ECOG = Eastern Cooperative Oncology Group; BMI = Body Mass

Index; SGA = Subjective Global Assessment; NLR = Neutrophil Lymphocyte

Ratio; PLR = Platelet Lymphocyte Ratio

The main observed any grade non-hematologic toxicities were alopecia (84%), nausea (49%) neuropathy (46%), weight loss (44%) and vomiting (40%). The most commonly observed any grade hematologic toxicities were anemia, lymphopenia, and leukopenia in 33, 31, and 30% of patients, respectively. No significant differences were found when relating toxicity and age. Male patients developed anemia (38 vs. 61.5; *p *= 0.001) and neuropathy (44 vs. 55; *p *= 0.03) more frequently than female patients did. Patients with poor functional status (ECOG 2) presented anemia (32.6 vs. 21.6; *p *= 0.001) more frequently than those with a good functional status (ECOG 0 and 1).

Patients who were moderately or severely malnourished and with hypoalbuminemia developed more chemotherapy-induced toxicity overall when compared with patients without malnutrition (31 vs. 22; *p *= 0.02) and normal albumin (54 vs. 41; *p *= 0.04), respectively. Hypoalbuminemia was associated with anemia (56 vs. 47; *p *= 0.05), fatigue (58 vs. 46; *p *= 0.01), and appetite loss (57.1 vs. 46.7; *p *= 0.004), in addition, it exhibited a tendency for the occurrence of nausea and neuropathy. Patients with diabetes showed a tendency to develop neuropathy (*p *= 0.08). PLR ≥ 150 was significantly related with the development of toxicity grade III/IV (59.3 vs. 47 p = 0.008). Other clinical and nutritional factors related with chemotherapy-induced toxicity are shown in Table 2. Overall toxicity development was significantly related with low albumin serum levels being more significant as the levels decreased (Table 3).

**Table 2:** Clinical and nutritional factors related to chemotherapy-induced toxicity development

	Anemia	Neuropathy	Appetite loss	Nausea	Lymphopenia	Fatigue	All toxicities	Toxicity Grade III/IV
Gender (Female vs male)	**0.001** **(38 vs 61.5)**	**0.03** **(44 vs 55)**	.851	**0.09** **(55 vs 46)**	0.9	0.5	0.5	0.5

ECOG (0,1 vs 2)	**0.001** **(32.6 vs 21.6)**	0.7	**0.022** **(45 vs 63)**	0.09	0.6	0.2	0.8	0.2

Diabetes	0.2	**0.08** **(51 vs 47)**	0.1	0.5	0.3	0.6	0.2	0.8

Albumin (>3.1 vs ≤3.0)	**0.05** **(47 vs 56)**	**0.06** **(36 vs 64)**	**0.004** **(46.7 vs 57.1)**	**0.07** **(48 vs 54)**	0.8	**0.01** **(46 vs 58)**	**0.002** **(43 vs 62)**	0.6

Weight loss (>10% vs <9.9))	0.3	**0.05** **(23 vs 30)**	0.4	0.7	**0.039** **(23.4 vs 30.9)**	0.3	0.5	0.2

BMI (≤19.9 vs >20)	**0.006** **(48 vs 76)**	**0.08** **(49 vs 68)**	0.4	0.8	0.2	0.2	1.0	0.6

SGA (well nourished vs malnourished)	0.1	0.7	0.7	0.2	0.7	0.3	**0.028** **( 31 vs 22)**	0.6

NLR (≥5 vs ≤5)	0.1	0.8	0.2	0.8	-	0.8	0.2	0.7

PLR (≥150 vs ≤150)	**0.004** **(37.9 vs 53.8)**	0.8	0.7	0.4	-	0.2	0.2	**.008** **(59.3 vs 47)**

ECOG = Eastern Cooperative Oncology Group; BMI = Body Mass Index; SGA = Subjective Global Assessment; NLR = Neutrophil Lymphocyte Ratio; PLR = Platelet Lymphocyte Ratio

**Table 3:** Global toxicity in relation to albumin serum levels before chemotherapy was given.

Albumin level	Mean ranks	P
≤ 3.8	38.5 *vs *53.3	0.046
		
≤ 3.5	41 *vs *54	0.040
		
≤ 3.1	43.8 *vs *62.2	0.002

## Discussion

Nutritional and functional deterioration are so frequently encountered in cancer patients that they are often accepted as part of the disease and its treatment [18]. The majority of patients with advanced NSCLC also present with malnourishment [31-34] and subsequently, with hypoalbuminemia. In our study, we found that one half of our patients were malnourished and presented hypoalbuminemia prior to chemotherapy. One of the most frequently employed first-line treatments for NSCLC is P plus TXN [35], both have been strongly associated with toxic side effects [36]. However, the potential benefits of this therapy in general outweigh the possible risks. The most common side effects of TXN used alone or in combination with other chemotherapy medications and demonstrated in some trials include anemia, leukopenia, neutropenia, alopecia, nausea, vomiting, and diarrhea [14-16].

The binding of TXN to plasma proteins such as albumin is important for achieving its therapeutic target. Albumin binds TXN and transports it in the bloodstream, allowing its gradual liberation, thus decreasing its toxic effects [37]. Therefore, we thought it would be useful not only for determining nutritional status, but also for predicting toxicity in patients with NSCLC under chemotherapy, given that albumin levels are not influenced solely by the oncologic process, but also by the patients protein and caloric intake prior to and during treatment. We found that patients who were well-nourished and with normal albumin levels benefited in terms of developing less toxicity after two cycles of TXN-P chemotherapy, on comparison with patients who were malnourished and had serum albumin levels below 3.0 mg/dL, mainly in the development of anemia, neuropathy, and nausea. In addition, with more severe hypoalbuminemia, major toxicities were found (Table 3). Serum albumin concentration has been traditionally used as a biochemical marker of nutritional status; it is one of the easiest parameters to measure and that which best reflects the state of visceral protein [38]. Although albumin is the main protein synthesized in liver [39], its long half-life and broad distribution in the body prevent nutritional changes from being reflected rapidly in albumin serum concentration [40]. Several processes control plasma albumin concentration, including absolute rate of albumin synthesis and fractional catabolic rate (FCR), among others. It has been previously demonstrated that patients with advanced cancer have an important energy expenditure caused by increased tumor metabolism [41] and specifically in patients with NSCLC, also by means of chronic airway limitation [42] Therefore, serum albumin levels can be modified by tumor activity via increased FCR, but also by decreasing albumin synthesis, given that the majority of the time, patients with NSCLC display decreased appetite [43] and diminish their food intake [44].

Currently, some studies have associated SIR with a poor prognosis in different solid tumors, particularly in lung cancer [40,45] The development of SIR in oncologic patients is unclear, which might be related to a tumor hypoxia-necrosis and local tissue damage causing disturbances in the neuro-endocrine metabolism, interleukine synthesis and acute phase protein production [46]. Clinical evidence has also shown that the activation of the SIR is one of the earliest and most important contributory factors of cachexia, moreover these findings help explain the failure of simple nutritional programs to reverse weight loss adequately in patients with cancer [34]. In our study, we used two frequently used parameters of measuring SIR, NLR and PLR [18,27-30].

Besides, albumin synthesis may diminish with SIR found in patients with advanced NSCLC [18]. We found a relationship between ≥5 NLR and baseline hypoalbuminemia and also of PLR ≥150 with baseline hypoalbuminemia and low BMI. Moreover, patients who had an initial PLR ≥150 develop higher toxicity grade III/IV and anemia. In previous studies, PLR and NLR confer an independent prognostic value in oncologic patients; an increase in absolute neutrophil and platelet count might be implicated in the development of methastases [47] and low lymphocyte levels could be related to a decreased cell-mediated immunity against tumor tissue [48]. However, these values have not been used as markers of chemotherapy toxicity. Thus the NLR and PLR might be potential biomarkers to predict whether a patient will develop toxicity in this regard because it is almost universally available and adds no additional cost to routine laboratory measurements. Clearly further validation work and a feasibility study are required before it can be considered for clinical use.

Although malnutrition has been associated with increased hospitalization, increased susceptibility to infection, reduced QOL, and increased mortality [49-51], nutritional status evaluation may not be afforded sufficient attention in the field of chemotherapy-induced toxicity in patients with NSCLC, who themselves represent a considerable risk factor for complications and co-morbidities. Albumin is habitually included among parameters utilized for nutritional assessment and has recently become more widespread [52]. It has also been established that serum albumin concentration was an independent prognostic variable for survival in 90 patients with NSCLC [53]. Notwithstanding this, to date few data are available in the literature concerning the prevalence and clinical significance of hypoalbuminemia in oncologic patients and the manner in which it affects their treatment.

Albumin levels, PLR and NLR were all related to the development of chemotherapy-induced toxicity. We therefore consider that the SIR, hypoalbuminemia and malnutrition in unison contribute to its further development.

## Conclusions

SIR parameters were associated with malnutrition, weight loss and hypoalbuminemia. Malnutrition and hypoalbuminemia are associated with the development of chemotherapy-induced toxicity in patients with NSCLC treated with P and TXN. Therefore, early nutritional assessment and detection of SIR markers might allow identification of patients at higher risk of developing chemotherapy toxicity and the implementation of an adequate nutritional support might be accompanied by beneficial effects when treating patients with NSCLC; in turn, this may permit completion of maximum oncologic therapy and improve treatment results with a more favorable toxicity profile.

## Abbreviations

NSCLC: Non-small-cell lung cancer; SGA: Subjective Global Assessment; TXN: Paclitaxel; QoL: Quality of life; ECOG: Eastern Cooperative Oncology Group; BMI: body mass index; CNs: Clinical Nutritionists; NCI CTCAE: National Cancer Institute Common Terminology Criteria of Adverse Events; SDs: Standard deviations; CIs: Confidence intervals; SIR: Systemic inflammatory response; NLR: Neutrophil Lymphocyte Ratio; PLR: Platelet Lymphocyte Ratio

## Competing interests

The authors declare that they have no competing interests.

## Authors' contributions

OA participated in the study design, carried out the statistical analysis, interpreted the data, and drafted the manuscript. RMMO conceived the study, and participated in its design and coordination, and revised the manuscript. GV participated in its design and coordination and critically reviewed the manuscript. GS, ST, CM, NO and LM participated in concept, design, data collection and writing. DF participated in the coordination of the study. KSL conceived the study, and participated in its design and coordination, and revised the manuscript. All of the authors have read and approved the final manuscript.

## Pre-publication history

The pre-publication history for this paper can be accessed here:

http://www.biomedcentral.com/1471-2407/10/50/prepub
